# Emotional distress among frontline research staff

**DOI:** 10.1016/j.socscimed.2021.114101

**Published:** 2021-06-05

**Authors:** Megan Nguyen, Lloyd Goldsamt, Nonhlanhla Mazibuko, Sanelisiwe Zondo, Rebecca Fielding-Miller

**Affiliations:** a University of California, San Diego Herbert Wertheim School of Public Health, USA; b New York University, USA; c Independent Research Consultant, Mbabane, Eswatini

**Keywords:** Research ethics, Global health, Researcher trauma, HIV, Eswatini, Public health

## Abstract

Public health research frequently deals with sensitive topics. A growing body of evidence suggests that frontline researchers who elicit or process participant’s traumatic experiences are themselves at risk of developing emotional distress or secondary trauma from daily immersion in these data. This both threatens a study’s data quality and calls into question how the harms and benefits of conducting research are distributed across a study team. The objective of this study was to explore how frontline research staff in Eswatini experience and process emotional distress as part of their daily work and to describe potential strategies for resilience and coping using qualitative research methods. We conducted 21 in-depth interviews with informants who had worked in data collection, data entry, and transcription on a number of sensitive topics, including HIV, sex work, and LGBT health. We found that emotional distress is a salient experience among frontline research staff working in Eswatini. This distress stems from conducting research against a generalized backdrop of high rates of HIV, violence, and poverty, particularly since research staff are drawn from affected communities and have their own firsthand knowledge of the phenomena they are studying. Moreover, the qualities study staff are often hired for – empathy, compassion, and emotional intelligence – are also traits that may increase their likelihood of feeling distressed by the narratives they encounter in their work. The workplace can serve as a prism, exacerbating or potentially mitigating these risks into harm at the individual, interpersonal, and community level. While not all study teams may have access to formal mental health services, several informants recommended incorporating regular meetings with a trained counselor as part of the overall project. Others recommended building time for team-building or debriefing conversations into the normal workweek, a strategy that would address both the issue of workload and could bolster the already existent strategy of relying on team members for mental health support.

## 1. Background and significance

By its nature, much of public health research deals with sensitive topics. A discipline concerned with measuring and improving the health of populations must necessarily focus a great deal of its attention on illness, mortality, and social disparities. While public health research is typically grounded in a biomedical paradigm, in which the project of research comes with an expectation of objectivity and professional distance (Tebes, 2005), the events of 2020 have made it increasingly clear that public health researchers have always occupied a dual role as human beings who are themselves susceptible to the morbidity and mortality which they seek to study as scientists. As the COVID-19 pandemic has altered daily life in nearly every country of the world, issues of burnout, fatigue, and compromised mental health among researchers as well as healthcare providers have come increasingly to the fore (Yong, 2004).

Best practices exist for conducting ethical research pertaining to traumatic experiences – particularly gender based violence (GBV) – to ensure that these studies do not endanger or re-traumatize research participants (Ellsberg and Heise, 2002; Fontes, 2004; Legerski and Bunnell, 2010). However, a growing evidence base suggests that study staff may also risk emotional distress when they engage in research on traumatic topics (Eriksen, 2017; McLennan et al., 2016; Kiyimba and O’Reilly, 2016; Dominey-Howes, 2015; Drozdzewski and Dominey-Howes, 2015; Calgaro, 2015; Klocker, 2015; Coles et al., 2010; Coles et al., 2013). Maintaining a stance of ‘objectivity’ can be particularly difficult for researchers working on these topics, and necessitates an extra level of reflexivity (10). The existing literature on the adverse impacts of processing trauma as “evidence” is small but multidisciplinary and calls attention to the role of researchers as human beings capable of trauma and harm, not just simply objective observers (Drozdzewski and Dominey-Howes, 2015). In global public health research, frontline research staff such as qualitative interviewers, survey administrators, and transcriptionists, are at heightened risk of experiencing vicarious or secondary trauma as a result of the emotional material which they must elicit, process, or transform repeatedly as part of their daily tasks (Kiyimba and O’Reilly, 2016; Coles et al., 2014).

Institutional review boards (IRBs) and other bodies that provide research ethics oversight are charged with ensuring that the potential benefits of exploring sensitive and traumatic topics in research outweigh any potential risks to study participants. However, few resources exist which consider how the risks and benefits of research are distributed between relatively socially privileged and well resourced PIs and the frontline research staff whom they hire to directly engage with participants’ narratives and representations of trauma. Anecdotally, many PIs do work to ensure that safety protocols and debriefing sessions are in place to protect frontline research staff^12^, however these are not required by IRBs or other ethics oversight bodies, nor do recommendations for best practices exist (Grundlingh et al., 2017).

The history of international public health research in particular is deeply intertwined with the history of colonialism (Connell, 2014). Recently, Connell, among others, have argued the imperative to consider the power differentials inherent in research executed in the global South, but conducted and framed by researchers who are largely based in the global North (Connell, 2014). This can be extended to apply to domestic research as well, given the typical differences in pay and social prestige between academic PIs and their frontline staff who are most likely to have daily contact with participants and communities. This framing calls on researchers to consider not simply individual level issues of respect, autonomy, and beneficence for those recruited as human subjects in research, but the broader differentials in power within study teams.

There is currently a lack of data on how frontline staff in low resource settings experience or develop emotional distress as a part of their daily work. There is also a lack of information as to how power imbalances between PIs and local study staff may affect frontline researcher’s willingness to self-advocate or request support when and if they do experience vicarious trauma or distress. Finally, very little research exists on the strategies which frontline staff use for coping or resilience, particularly in contexts where conversations about mental health and formal mental health resources are limited. This study aims to qualitatively explore emotional distress among frontline research staff in Eswatini, a small country in southern Africa formally known as Swaziland, to understand (Tebes, 2005) Swazi researchers’ experiences and perspectives on working with trauma-related research materials (Yong, 2004), Strategies for resilience or coping, and (Connell, 2014) How power imbalances within study teams may exacerbate the potential for secondary or vicarious trauma in public health research.

## 2. Methods

### 2.1. Setting

Eswatini is a small absolute monarchy in sub-Saharan Africa with the highest HIV prevalence in the world (DemographicHealth Survey, 2007).^.^ As a result of the high prevalence of HIV, the nation hosts a great deal of international research within its relatively small borders. Eswatini has high unemployment rates – approximately 30% of Swazis do not participate in the formal economy, with even higher rates among women and young adults (Kingdom of Eswatini Count, 2013) – and local study staff typically work on short-term contracts as interviewers, survey administrators, recruiters, or transcriptionists, often rotating from study to study as opportunities become available. Formal mental health resources in the Kingdom are scarce. In 2011, there was one state psychologist who was available to provide services for study staff working on projects in which the national government was an active partner (This information was prov, 2010). As of 2017, there was one psychiatrist and approximately 4 trained psychologists working in either the private or public health system for a nation of approximately 1.1 million (Global Health Observatory, 2019).

### 2.2. Approach

This was a qualitative study utilizing in-depth interviews. The project was led locally by two study coordinators (SZ and NM) who both have several years of experience conducting qualitative research related to HIV and gender-based violence (GBV) in Eswatini. RFM (the senior author) has conducted research in Eswatini since 2010 and has a large professional network of Swazi and expatriate colleagues with experience conducting research on HIV, GBV, and research with marginalized populations in the country. SZ, NM, and RFM have known one another for approximately a decade, including collaboration on a previous study conducting qualitative research on sex work and food insecurity in Eswatini (Fielding-Miller et al., 2014).

We began by reaching out to research colleagues in Eswatini and asking them to refer us to potential participants. From these initial contacts we then continued to recruit participants using an iterative, snowball-sampling approach. We attempted to interview frontline researchers with a diversity of experiences by research topic, age, and gender. NM and SZ conducted and transcribed the interviews. All interviews were guided by a semi-structured field guide whose domains included participant work history, common local terms for experiences of emotional distress, local mental health schemas, personal experiences of distressing research encounters, personal strategies for resilience and coping, and barriers and facilitators to help-seeking. Interviews were recorded and transcribed verbatim and analyzed iteratively. RFM, SZ, and NM met regularly either in person or over WhatsApp to discuss emergent themes and refine the guide as necessary. Recruitment continued until the study team agreed saturation had been reached. We then conducted a basic thematic analysis of all transcripts, guided by the social-ecological model to organize the experiences and determinants of emotional distress among frontline researchers at the individual, interpersonal, community, and structural levels (The Social-Ecological Mod, 2021).

### 2.3. Ethics

All participants provided informed consent. We used oral consent to minimize confidentiality concerns given the extremely small and closeknit nature of the Eswatini research community. Participants received E50 (approximately $3) to thank them for their time and expertise. This amount was based on a typical fair hourly wage for research assistants in the country at the time. All interviews were transcribed by SZ or NM to reduce the risk of confidentiality breaches. We shared preliminary results with research participants, government research bodies, and local and regional branches of international research organizations. The research protocol was reviewed and approved by the University of California, San Diego Institutional Review Board and the Eswatini Scientific Ethics Committee.

## 3. Results

**Fig. 1.**
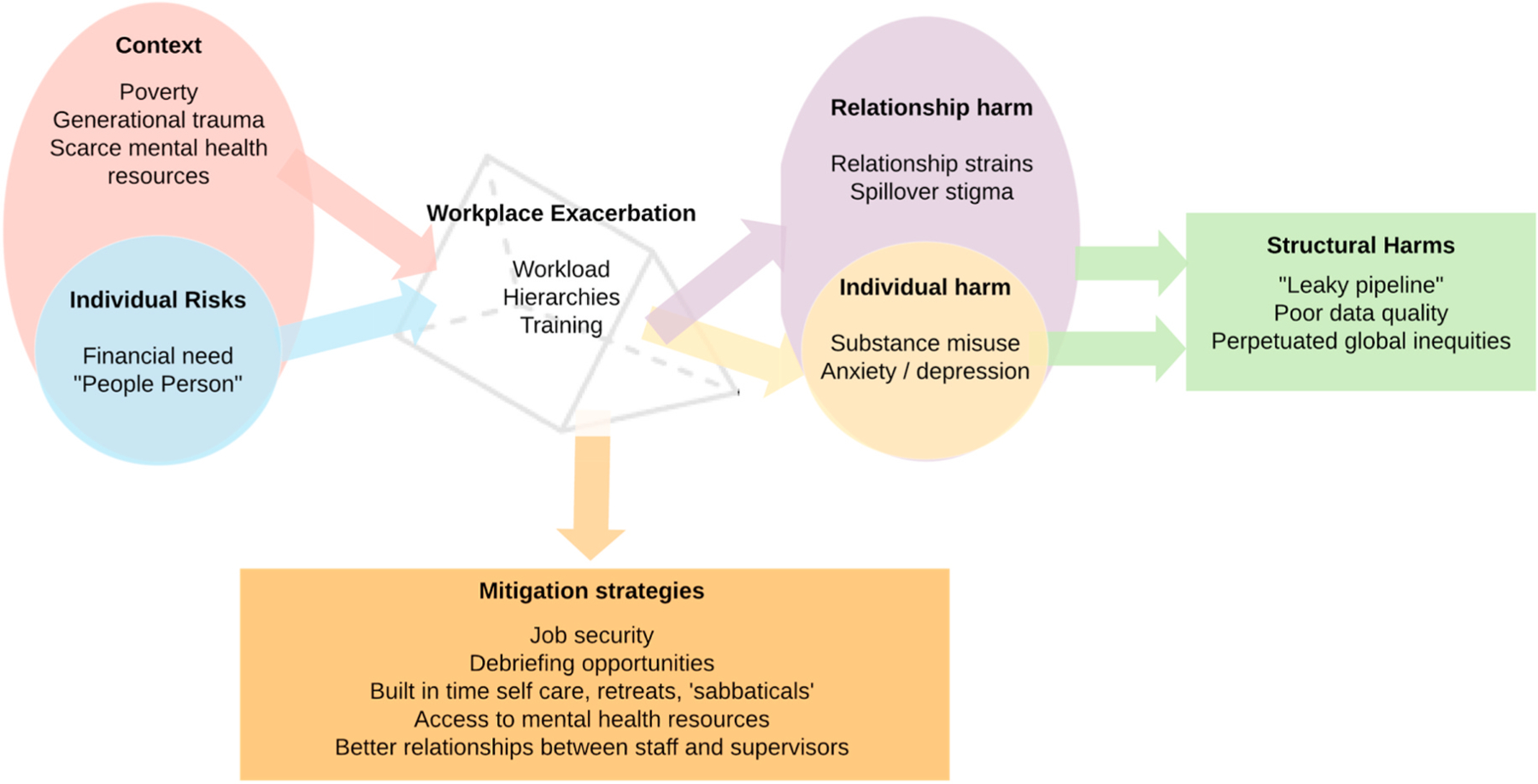
Conceptual framework of qualitative findings illustrating how individual and structural level risk factors puts frontline research staff at heightened risk of emotional distress in the workplace. The workplace functions as a prism that can reflect risk factors out to create individual, relationship, and structural level harms, or the workplace can adopt various mitigation strategies to prevent additional harm.

We conducted interviews with 21 informants, 8 women and 13 men. Participants had worked in data collection, data entry, and transcription on a wide variety of topics including cancer, HIV, child abuse, sex work, violence, and LGBT health. Across these interviews, participants described both individual and structural level risk factors and harms that manifested at the individual, relationship, and community level. The workplace emerged as a key site with the ability to either exacerbate or mitigate these factors and participants shared specific recommendations that study team managers could put in place to address these (Fig. 1).

## 4. Risk factors

### 4.1. Context

Participants repeatedly discussed the mental health toll of doing daily work on sensitive subjects against a broader social backdrop of poverty, HIV, violence, and stigma. Frontline research staff explained that maintaining professional distance or mental boundaries was extremely challenging in this context because they often had deeply personal experiences with these same topics on which they were hired to gather data. Swazi communities are closely knit, and it is cultural anathema to ignore a neighbor in need when one has the resources to help. Many of the frontline staff we spoke with felt deeply moved by the living conditions of the people whom they met while engaged in fieldwork. Several reported that they found it distressing to listen to these stories as part of their daily work.

“I remember there was this homestead I went to, it belonged to a grandmother who lived with her grandchildren. When I introduced myself to her, she pleaded that I help her by taking her granddaughter. She said the child’s parents both succumbed to AIDS and she cannot support the child. She pleaded for me to take her in order to help her ease the burden … that’s one of the many emotionally draining experiences”.

- (ND2, Evaluation Officer).

Frontline researchers reported that while they might welcome professional help to cope with the emotional distress brought on by their work, it is not currently culturally normative to seek professional mental health care, nor are the resources available for individuals who would wish to do so.

“So the issue with psychosocial support even our government is dragging her feet on that I would say, because if you can look at the number of psychologists employed by our government you find that there is one here in Hhohho, one in Lubombo and one in the other regions. Even the information concerning where you can get psychological support it’s not there. So now not everyone gets access to that service … So I would say it’s a gap that exists and our government needs to [encourage] awareness that there is help here and you can go to the clinic for mental health and a counselor can help you” (ND1, Adherence counselor).

### 4.2. Individual risk factors

Multiple frontline study staff described themselves as a “people person”, and felt proud of their ability to listen to study participants nonjudgmentally and empathetically - key skills for data collectors working on sensitive subjects. However, the same empathy and emotional intelligence for which study staff were often hired, and which they were encouraged to cultivate in their job training, also appeared to put them at risk of increased emotional distress. Many explained that the stories they collected in their work could linger for a long time after fieldwork, data entry, or transcription was complete.

“For instance, when dealing with a rape case, as soon as the person touches on that topic, obviously you will react or feel sorry for the client. As a human being you will be touched emotionally. You will find that this person contracted HIV through rape. The first thing that comes to mind is your own children and can’t help but imagine how it would be if this was my own child undergoing this situation” (Jukes, Counselor).

I am a loving person, I love children and if I see them suffer then that hurts me. I tend to imagine that what if this is my own child. What would I do?” (Kim, Data Extractor).

Informants also reported feeling intense guilt when they were unable to provide direct relief to the people whom they met in their daily work. Many grappled with what to do when the limit of their responsibilities as members of a (foreign funded) research team came into conflict with their desire to help other members of their community. Some tried to take comfort in the hope that the data they collected could potentially inform future interventions that would benefit their community in the long-run. One informant (Miso, Interviewer), explained:

“It’s really painful knowing that there is nothing you can actually do in order to help this person. Like when they get to tell you their stories somehow they are crying out for help they think you will help them. But then you comforted by the fact that these programs which will be established with their findings will help them in order to make their work much safer.”

Not every frontline staff member we spoke with was optimistic, however.

I would tell them that the Ministry of Health will try their level best to help better this situation but deep inside you, you are aware that this will not improve … and that is disturbing because you are making empty promises yet in research you are not supposed to give out false or misleading information … but then you feel the need to reassure the people that one day all will be fine. (Kim, Data extractor).

While study staff were initially drawn to the work because they identified as empathetic, “people persons”, the long hours, chronic feelings of distress, and frustrations of the work often left them feeling jaded, disconnected, or generally burned out by their jobs. When given the opportunity to voice their concerns to the US-based research funder of the study, participants highlighted the complex tension of research funding from the global North simultaneously providing both help and harm: a source of temporary income and real emotional damage that many felt unable to leave due to high unemployment.

**Interviewer:** What you would like the people of the US to know about the type of work that you do?

**Respondent:** I would like to thank them for providing employment opportunities for Swazis [so that we can] put food on our plates, [although] our job does drain [us] emotionally and we are hoping that this study will help us.

### 4.3. Workplace exacerbation

The workplace functions as a prism through which individual and structural level risks has the potential to be transmuted into individual, interpersonal, or community level harms. This process primarily functioned via relationships between staff, supervisors, and international members of the study team. Some study staff had worked on projects in which team members met for regular debriefings. Individuals who had had access to this opportunity felt that this was an effective strategy for dealing with distress. When asked about why she shares her feelings exclusively with her colleagues, one informant responded:

“Okay the ethics part that how can I say this okay it felt bad but then you know that when you experience something sometimes you need to vent out something you may have gone through … okay it was just to find out that is it just or they were also going through similar thing because sometimes you know you also draw comfort from other people who go through similar things. You know just to hear that you are not the only person who is going through similar things. Also sharing with them helped me as it gave me better understanding, the different places we go to there was something unique about those places. So you get to gain a better understanding of why certain things happen at this particular area” (Zee, Interviewer).

While for some informants debriefing was an important protective factor against emotional distress, others discussed how restrictive workplace cultures and poor relationships with direct supervisors left them unable to “blow off steam.” These informants felt that they were treated primarily as research instruments, rather than as human beings with real feelings. One (Zee, 23, Research Assistant), described feeling “looked down upon” and treated like “cheap labour”, regardless of any prior research experience or professional qualifications.

Several participants reported being unable to discuss their feelings with their supervisors, not only because they believed their emotions would be disregarded, but also out of fear of being punished with lower pay or threatened with unemployment. Supervisors were described as unsupportive, unapproachable, and solely concerned with work and money. While for these supervisors the emotional toll was normative, it also did not warrant any staff accommodations. Sam (NGO worker) explained:

“Those people are very difficult, unapproachable that the first thing about them. The only thing they care about is their work just like we do with money. So the only time you can talk to him/her is the time maybe you are unable to log in on some application. If it’s work related then you can talk but then if its personal thing then they simply tell you that we hired you since you said you are flexible to do this. They simply tell you that their hands are also full with their personal problems. They are not even willing to listen they are always busy.”

Multiple informants described experiencing a great deal of emotional distress due to what appeared to be misinterpretations of the role of research subject confidentiality. Many study staff perceived ethical guidelines around confidentiality to mean that they were prohibited from discussing any element of their work with any other person, including their colleagues, even in an anonymized way. They feared that doing so breached the principles of research ethics. One informant explained why the confidentiality clause acted as a source of distress:

“I can also not tell them because I feel as a researcher I am prohibited by the confidentiality clause that I should have … I can’t be spreading things […] That’s why I choose to […] put this emotional burden on myself” (Shaz, Researcher).

Nearly all informants discussed struggling with large workloads, which directly contributed to or exacerbated their distress. Informants cited strict deadlines, the need to meet specific interview quotas to be paid sufficiently, and long, difficult interactions with research participants as particularly challenging and draining tasks involved with research. Study staff pointed out that many times these constricted deadlines were a direct results of delays in the overall project that were beyond their control, however they were still expected to collect data on a rapidly accelerated timeline to meet original deadlines.

“The questionnaires are too long and you have to do as many interviews as you can in order to net a lucrative salary at the end of the month. Now you have to work long hours in order to make money. Sometimes you have to work when meeting your respondents. Like with the last research project I working on. We used to visit awkward places where cars cannot reach. So they dropped us at a station then your walk to that particular home. At the end of the day you end up visiting view homes. So we also have to look out for ourselves and not to over work our bodies. Money you have it now and tomorrow it’s gone (Mzwandile, Data Collector).”

At the same time, being paid late was common to the point of being considered normal, albeit frustrating. Frontline staff had little recourse when they were paid late or inconsistently, in large part because they were generally hired on short-term contracts with few opportunities for upward mobility.

These NGOs [non-government organizations] ask for funds for a particular project […] they stipulate a specific or estimated number of people they are going to hire, in their proposal, whereas on the ground, they do not do that. Once they get the funds they hire very few people and task them with a lot of duties, yet they received huge amounts of money for the project. So they are the only ones who actually benefit from the project. The employees end up with loads of work. When advertising for posts, they mention that they need people with degrees. When they have hired their stuff however, they do not pay them based on their qualifications. They will make excuses about the funds when they actually received more than enough from the funders […] Yes, in you contract, it would be clearly stated by them that you will be earning a salary of about E9 000 but when end of month comes, they will give you E6 000. When you try and confront them about the salary issue they will tell you to wait until the end of the study for the money owed (Nokuthula).

Multiple frontline study staff described how the need to meet rigid interview quotas on short deadlines had a negative impact on the data collected. Several of the frontline staff we interviewed described witnessing other members of their study team fabricating study results:

“The first challenge, the working hours are long since you have targets and deadlines to meet. So now you have to push your work as much as you can and mind you are getting pain ion deliverables so if you have conducted few interviews so it means you won’t earn much. This also tend to jeopardize study results as some researchers end up filling the questionnaires for themselves, the reason which can make one end up doing such is that people sometimes tend to be difficult. Like you call them prior you schedule for an interview and you agree on time and day. When the day of the interview comes you can them to confirm now they will tell you that they are no longer available. It’s a short notice and you cannot just call another client and ask to interview them at that very same time. So now you have to do more interviews in order for you to get a better pay cheque” (Miso, Interviewer).

### 4.4. Harm

Harm manifested in staff’s lives at the individual, relationship, and community level.

### 4.5. Individual harms

Although we did not specifically ask about substance use, five participants spontaneously mentioned concerns about their own misuse of drugs or alcohol, or what they had witnessed among colleagues. Several participants discussed how their inability to release pent up workrelated frustrations forced them into more dangerous, higher-risk coping mechanisms.

Some people they tend to confide to their close friends but some they don’t, they tend to find solace in drugs like drinking alcohol and smoking dagga [cannabis]. These are the common drugs they tend to. With some they become suicidal but such cases are not common. For those who tend to drug abuse is those people who feel they don’t have anyone to tell their problems or to seek comfort. Like they feel that if they do talk to anyone about their problems they will go around telling everyone and they will be everyone’s cup of tea. So they keep it to themselves and try to deal with their problems on their own whereas this is a mental health hazard. It can lead to one in becoming mental unstable” (Miso, Interviewer).

Many participants linked a lack of debriefing opportunities to poor mental health among frontline staff. This could present in many ways. Several frontline staff described instances in which they felt depressed, anxious, or suicidal. These more intense reactions were rare, although several informants did describe having felt that way themselves in the past or knowing other study staff who had felt that way. Most common were feelings of intense sadness and irritability.

**Interviewer:** In general, what do people here in Eswatini do when they have emotional experiences like the ones we’ve been talking about?

**Respondent:** Umm, they react differently so they become angry, some don’t want to talk and soothe their feelings through drugs abuse, some end up committing suicide yah because of depression and anxiety” (Mntana, Interviewer).

### 4.6. Relationship harms

While nearly every participant named social support as a key means of addressing work related distress, the same work had the potential to severely damage those same social support systems. Long hours of data collection took a toll on study staff’s relationships, and this could be compounded if staff members felt anxious, irritable, or angry, or withdrawn (common symptoms of depression). One focus group facilitator explained why she ended her relationship with her boyfriend because of her work:

“It affected my relationship with my boyfriend because he felt I spent too much time at work. And because of the emotional distress, I would sometimes not want to talk to him or see him because of depression, and he thought my work was more important to me than him … I broke up with him. Because I’m dedicated to my work, and since he failed to understand that, it wouldn’t have worked.”

Several informants reported relationship strain due to the stigma associated with their work and its involvement with key population. Informants came into conflict with their families in particular who knew of their work with highly stigmatized populations. Other informants tried to avoid disclosing the exact nature of their work to their friends and family, leading to damaged relationships. Several reported experiencing “spillover” stigma as a result of their work. Study staff often confronted rumors that they too were members of a stigmatized community regardless of their individual sexual orientation or work histories. While the experience of stigma was not as severe as that experienced by men who have sex with men or female sex workers, these rumors could still damage study staff’s relationships and their reputations in their community. Several reported that they simply did not share the details of their work with family and community members to avoid any potential social repercussions. As one data collector explained when asked what friends and family thought about her job:

I think they reserve their comments because maybe they have negative thoughts about my job … Because of the nature of my job and many people assume that when you work at an HIV/AIDS organization then you are HIV positive yourself …. It is not nice to be judged based on an illness. The fact that they think you are HIV positive is not nice”

(Chrissy, Research Volunteer).

### 4.7. Recommendations

Research work in Eswatini is typically conducted on short-term contracts, and the staff we spoke with had worked on a number of different projects across their research careers. They repeatedly emphasized the need for permanent and secure job opportunities. A significant portion of study staff distress was attributed to the persistent threat of being unemployed when short-term contracts came to an end.

“What I can say is that our job doesn’t provide with security. It’s sessional like working for a few months or years then you are unemployed. I wish there was a way for this job to be on permanent basis. Like myself I don’t see myself doing any other job besides research” (Mzwandile, Data collector).

Participants nearly universally recommended regular workplace debriefings for those engaged in research on sensitive topics. Participants discussed how they would like to see structured meetings in the workplace where they could vent to fellow research staff who could relate to their experiences. Immediate supervisors can play a key role in both creating the space for these meetings, and creating a workplace culture in which employees feel safe sharing their vulnerability and distress:

**Interviewer:** Ok so what can supervisors do to make sure the research assistants stay emotionally healthy and supported?

**Respondent:** Have briefing and debriefing meetings. They should find out what we are going through as research assistants. They should find means to address stressful issues.”

The relationship between research staff and supervisors was a crucial facet of addressing work-related distress. Specifically, respondents felt that supervisors “must take care of their RAs [research assistants]” (Cebosh, Data collector). Participants suggested that supervisors were primarily responsible for providing an effective working environment by being understanding and sympathetic, and respectful.

“They should be gentle and understand that we also human. That when we get there out in the field, that we are at work but we will be touched by the incidents these people go through. Let us have a stage that when we come back and share that this and that is what happened and this is what my experiences were like. While stile still out in the field which things didn’t treat you right or which things went alright”

(Shaz. Researcher).

Many of our informants pointed to the ways in which workplace hierarchies further exacerbated harms. These hierarchies flowed from the (typically) international PI, and several of our study participants expressed frustration with the way their labour was valued compared to the supposed value of the project, as well as their perception of how funds were allocated across project elements.

“They just treat you ahhh … even if you have a BA, they treat you like you are needy. They will make you do whatever they want … They don’t consider that it’s the weekend so stay at home, because they know that there is no other job. The chance you got for this research [job] you will accept every situation handed to you and all their conditions”

(Shaz, Researcher).

“We know that Americans donate lots of money for such projects but then it tends to be misused by those who are in power, like us research staff we work hard but we earn peanuts. You know you even work extra hours but you are not paid for your overtime”

(Miso. Interviewer).

“One they should know that we are very good at what we do. But then it’s all about the people that get the tenure … you finds that some of them they exploit us and I feel like … I don’t know what Swaziland can do … There’s not much except for we do our jobs perfectly and we are being exploited. More than anything we are being exploited”

(Mzwandile, Data Collector).

Other participants highlighted the need to value each team member equally, and clearly linked disrespect in the workplace to increased harms of research work.

“They should be friendly and not look down upon ones in other positions at the work place. Even if the person is a cleaner. So like us I felt we were looked down upon by the organisation we were working with. They didn’t consider us as part of their staff … So supervisors they need to be friendly in order for everyone to easily approach them when they have issues pertaining their work” (Mntana, Executive director of health).

While it is not common or culturally normative to access clinical mental health care in Eswatini, several participants discussed how access to a psychologist or counselor would be beneficial to study staff working on emotionally difficult projects.

“I think they [professional counselors] are very much needed by researchers. When I am stressed and I can’t even open up to my supervisor or the next person because I feel like they will be judgmental. When I get to the counselor I can be able to open up completely without leaving anything out and can be given better solutions because this person is not part of our team and doesn’t go to our field of work”

(Chrissy, Clinical data extraction).

A few informants specifically suggested that frontline research staff be provided opportunities for vacations and retreats in order to “refresh their mind” (Rock, Research assistant). Informants discussed how time for vacation and retreats would allow them to distance themselves from their work and focus on their well-being. They described these opportunities as potential “de-stressing tools”

(Sam, NGO worker).

“Retreats are also needed. Maybe once in three months, for example go for a week’s retreat to Lugogo to refresh. Out there you will meet new people and learn more from them and they will learn something from you” (Jukes, Counselor).

## 5. Discussion

We found that emotional distress is a salient experience among frontline public health research staff working in Eswatini. This distress stems from conducting research against a generalized backdrop of high rates of HIV, violence, and poverty, particularly since research staff are typically drawn from affected communities and are likely to have experienced or know someone who has experienced the phenomena they are studying. Moreover, the qualities study staff are often hired for – empathy, compassion, and emotional intelligence – are also traits that may increase their likelihood of feeling distressed by the narratives they encounter as part of their work. Our findings suggest that the workplace can serve as a prism, exacerbating or potentially mitigating these risks into harm at the individual, interpersonal, and community level. Study staff we spoke with recommended building in opportunities to debrief at work, more access to mental health resources, greater attention to equity and respect across the whole study team (including the primary investigator who is typically based at a North American or European institution), improved job security, and more opportunities for self-care.

The equitable distribution of harms and benefits across communities is a core component of medical research ethics, however our findings describe the ways in which frontline staff seem to shoulder a disproportionate amount of the risk of conducting research, while the social and economic benefits largely accrue to study leadership. We cannot ignore that these risks and benefits follow historic colonial lines where leadership in the global North has the ability to control funds and establish study protocols and frontline staff largely based in the global South have little power to negotiate for better pay or working conditions, or to communicate potential issues with data integrity back from the field site to study leadership.

Study staff – particularly quantitative survey administrators and qualitative interviews – are often recruited directly from affected communities. Doing so allows data collectors to quickly gain entrance into communities and build rapport with individual informants, improving both the speed and quality of data collection. However, this same practice increases the likelihood that study staff are collecting data on phenomena that they themselves have also experienced firsthand and with which they may be intimately familiar. Study staff’s understandable difficulty distancing themselves from their research subjects and approaching traumatic material from a place of objectivity leads to a direct risk of retraumatizing or triggering staff (Dominey-Howes, 2015; Drozdzewski and Dominey-Howes, 2015). Staff who enter, clean, or analyze data are not immune from this, and transcriptionists are likely at even higher risk because their work involves repeatedly hearing a narrative of trauma or distress without the ability to intervene. In addition to the risks to the individuals serving in these positions, these experiences of trauma or emotional burnout can also pose a real risk to the validity of the data collected. When staff lose the ability to engage empathetically with study participants, or a project has high turnover as a result of burnout and disengagement, the research enterprise itself is likely to suffer. The threats to data validity are important in and of themselves, however researchers who are engaged in research related to traumatic subjects such as HIV or gender-based violence have a particular ethical obligation to ensure that their research is scientifically sound and actionable. If not, it becomes ethically problematic to request these narratives of trauma from study participants in the first place (Jewkes et al., 2012).

Rigid, institutionalized workplace hierarchies make staff feel unvalued and expendable by their superiors. This unequal power dynamic cannot be disentangled from public health’s history as a colonial enterprise with extractive practices (Beckham et al., 2015) that can magnify or reinforce existing inequalities between study team leads from the global North and frontline staff from the global South, even as the research itself purportedly seeks to address the health disparities which arise from these same historic forces (Spini, 2018). Young researchers may leave the field after negative or traumatic experiences early in their career, particularly if they’ve felt exploited, undervalued, or traumatized by their work with no institutional attempts to mitigate these. This loss of entry-level research staff is compounded by a lack of training opportunities and upward mobility, leading to a ‘leaky pipeline’ in which vital voices and first-hand perspectives are lost from the research enterprise. This same process compounds ongoing inequities, in which research leadership is most frequently vested in PIs from the global North who have more seniority within funding systems, academic credentials, and access to institutional support, and frontline study staff from the global South who are more likely to be early in their career and have less access to formal academic training.

Our findings suggest the necessity of integrating a collective or workplace care model into study methodologies, rather than simply encouraging self-care among study staff. Study team leaders must recognize that staff will have personal limits that will likely vary by topic depending on individual personal history. Moreover, staff must have the space to connect and build community to ensure the workplace is safe and supportive (Horn, 2019). To protect both the well-being of those engaged in front line research work and the research itself, the burden of responsibility to provide care needs to shift from individual staff members to workplace supervisors and superiors. Self-care is typically framed as individual actions taken to alleviate the burden of personal stressors. However, when the workplace is the cause of harm, it is reasonable to expect that the workplace should also take on the responsibility of mitigating that harm. The promotion of well-being should be taken on as a collective strategy where a healthy and nurturing environment is created within the workplace rather than as an additional professional burden placed upon individuals to address in their personal lives. Some of the suggestions provided by informants included opportunities for institutionalized debriefing and paid-time retreats.

Additionally, workplaces should provide opportunities for upward mobility and capacity building for frontline staff, who described shortterm employment contracts as one of their significant sources of emotional distress. Workplaces should direct their focus towards retaining those already employed and developing their staff’s skills for as long as possible. Doing so would alleviate frontline staff of their distress concerning potential unemployment and promote continuity in research. These opportunities for upward mobility and capacity building would also allow for workplaces to shift more power from the wellresourced study leadership of the global North to the hands of frontline staff in the global South. As Connell and others suggest, the future of social sciences relies on the integration of Southern perspectives to overcome the historically colonial tendencies of public health research (Connell, 2014). Empowering frontline staff as valued collaborators and granting them increased autonomy over their own work is a necessary step forward towards confronting power imbalances within study teams and global disparities in opportunity.

The current study is not exempt from these complex dynamics. SZ and NM are Eswatini-based researchers, while MN, LG, and RFM are based in the Global North and represent a range of institutionalized academic power as a student, junior, and senior faculty, respectively. While the goal of the study was to name and explore the power differentials inherent in North-South public health research, we recognize that our position and privilege as researchers from the global North and our reliance on our Swazi colleagues to collect data from their own community on topics that have affected them personally in many ways replicated the very power dynamics and neo-colonial power structures described above. We attempted to work from a framework of ‘solidarity’, rather than ‘aid’ (Frenk et al., 2014) by acknowledging and naming these differences throughout the process of study design, data collection, and analysis. The study was grounded in earlier conversations between SZ, NM, and RFM, based on their previous work together collecting qualitative data on food insecurity among female sex workers living with HIV in Eswatini (Fielding-Miller et al., 2014). SZ and RFM consulted during project conceptualization, and SZ, NM, and RFM discussed strategies for data collection and analysis at multiple stages throughout the project. SZ, NM, and RFM checked in regularly over WhatsApp throughout the process of data collection and analysis both to discuss data collection and to debrief about any potentially triggering incidents. SZ and NM also co-presented our initial study findings at a large global scientific meeting.

Our findings suggest that grounding the current study in solidarity, rather than charity, and working to ensure an equitable distribution of professional and financial resources across our team not only improved the rigour of our findings, but also align with an ethical imperative for researchers from the global North to pay close attention to how the harms and benefits of conducting research are distributed across study teams, from study leadership to frontline staff.

## 6. Conclusion

The experiences and needs of emotionally distressed research staff in low resource countries highlights the exploitative nature of global health research that reinforces structural differences in power and privilege. The comparison of researchers between the global North and the global South provides a framework for understanding the uneven distribution of harm and benefits between the two. IRBs and other research ethics oversight bodies do not currently take into consideration the disproportionate amount of risks that local staff drawn directly from the intended research communities are exposed to, as opposed to their more privileged counterparts. These ethical challenges in global health research calls us to be more thoughtful about the burdens of research work on frontline research staff and the safeguards that can be implemented in the workplace to temper the worst effects of research work. Supporting self-care practices in the workplace and focusing efforts on leveling unequal power dynamics within study teams are necessary steps to take to protect frontline staff. Further research on the experiences and needs of frontline research staff in low resource countries is needed to address this imbalance of harms and benefits in global health research and provide more equitable and beneficial working conditions for frontline staff.
